# TRENDS AND SOCIOECONOMIC INEQUALITIES IN DEMENTIA RISK IN CHINA, 2011–2020

**DOI:** 10.1093/geroni/igae098.0690

**Published:** 2024-12-31

**Authors:** Liying Luo, Liyun Liu

**Affiliations:** The Pennsylvania State University, University Park, Pennsylvania, United States; The Pennsylvania State University, University Park, Pennsylvania, United States

## Abstract

China faces a rapidly aging population. However, there lacks evidence about if the prevalence of dementia risk increased over the past decade and what biopsychosocial factors may have contributed to the trends. Using the 2011-2020 data from the China Health and Retirement Longitudinal Study (CHARLS), this study finds the first evidence of significant decreases in the prevalence of high dementia risk—determined as scoring 4 or lower on 25 items of the Telephone Interview for Cognitive Status—in China between 2011 and 2020. The decrease was more pronounced among older Chinese women. We then use the Kitagawa-Oaxaca-Blinder method to quantify the contributions of a set of socioeconomic and health variables to the differences in the prevalence of high dementia risk between the periods 2011-2013 and 2018-2020. The decomposition results suggest that for both Chinese men and women, the compositional reductions in illiteracy and low education are a major driver of the decline in dementia risk between the two time points. Changes in other socioeconomic and health risk factors play a relatively smaller role, although for males, the heightened risk of dementia due to smoking exerts an upward pressure on dementia risk trends. Our findings extend the literature by providing evidence that increasing education is an effective strategy for reducing dementia risk at the population level.

